# Classroom‐Integrated Kettlebell Training: Effects on Motor Performance, Attention, and Health in Primary School Children

**DOI:** 10.1111/josh.70136

**Published:** 2026-03-19

**Authors:** Nicole Braun, Romina Ledergerber, Eric Lichtenstein, Lukas Nebiker, Ralf Roth

**Affiliations:** ^1^ Department of Sports, Exercise and Health University of Basel Basel Switzerland

**Keywords:** pediatric health, school‐based program, strength training

## Abstract

**Background:**

Declining physical fitness and rising obesity in children call for effective interventions. Integrating kettlebell training into the classroom may provide a practical way to overcome existing barriers. This study examined its feasibility and effectiveness on second graders' motor performance, attention, and health.

**Methods:**

Three classes (pre *n* = 61; post *n* = 47) performed daily 15‐min kettlebell training for 7 weeks, while two classes (pre *n* = 33; post *n* = 31) served as controls. Pre/post assessments included mid‐thigh pull, grip strength, countermovement jump, sprint, shuttle run, eye–hand coordination, side hop test, Flanker task, body composition, and blood pressure. Linear mixed models analyzed group differences, reporting Cohen's *d* and 95% confidence intervals (CIs).

**Results:**

Small effects were observed in sprint (*p* = 0.01, *d* = 0.46, 95% CI [0.20–0.73], Δ = +3.6% [1.5–5.6]) and mid‐thigh pull (*p* = 0.14, *d* = 0.28 [−0.08 to 0.64], Δ = +5.5% [−1.7 to 12.6]).

**Implications for School Health Policy, Practice, and Equity:**

Fun, safe, and regular sessions with simple routines and age‐appropriate exercises are required.

**Conclusion:**

This classroom‐integrated program is feasible and effective, particularly for enhancing lower‐body strength and promoting movement during the school day.

**Trial Registration:**

NCT06910085

## 1 Introduction

Childhood overweight and declining physical fitness are increasing public health concerns [1, 2]. Nearly one‐third of children aged 7–9 years are overweight, particularly among those from low socioeconomic backgrounds [1, 3]. At the same time, key health indicators such as cardiorespiratory endurance and muscular strength have steadily declined [4, 5, 6]. As overweight children are likely to remain overweight into adulthood, their risk of chronic conditions such as type 2 diabetes, cardiovascular diseases, and hypertension is increased [7, 8, 9]. Insufficient physical activity is a major contributor, with two‐thirds of European children failing to meet the recommendations of at least 60 min of daily moderate‐to‐vigorous activity, including strength training [10, 11]. The transition to school further exacerbates inactivity due to prolonged sitting, which negatively affects physical and cognitive health [9, 12, 13, 14, 15].

School‐based interventions effectively improve physical activity, cardiovascular health, and muscular strength and reach children from all socioeconomic backgrounds [3, 16, 17, 18]. However, logistical barriers often limit their implementation [18, 19]. To address these challenges, our study implemented daily 15 min kettlebell‐based strength training in the classroom. Strength training in children is a safe and effective way to improve strength, body composition, and cardiovascular health [20, 21, 22]. Even brief school‐based activity interventions have shown positive effects on BMI, daily activity, and academic performance [23, 24].

This study aimed to evaluate the feasibility and effectiveness of daily classroom‐integrated kettlebell‐based training in second‐grade children, hypothesizing improvements in physical performance, cognitive function, and health‐related factors.

## 2 Methods

### 2.1 Participants

**FIGURE 1:**
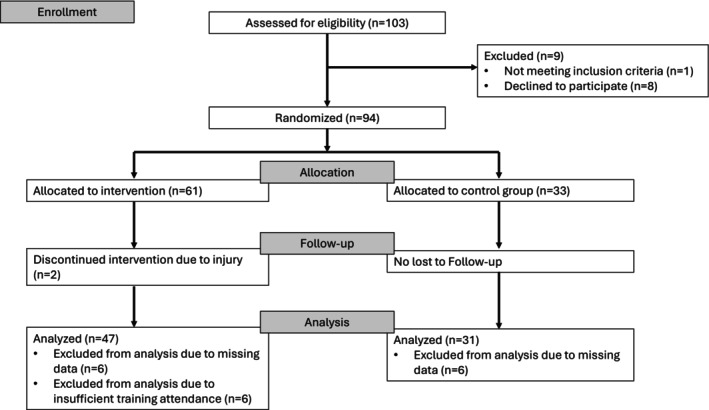
Flow diagram of participant recruitment and study procedures. *n* = number of children.

Second‐grade children in good health were eligible to participate. The study complied with the Declaration of Helsinki and was approved by the regional ethics committee (Ethics Committee Northwest and Central Switzerland; trial registration: NCT06910085). Of 102 invited children, 93 participated after obtaining written parental consent, with eight declining or not responding and one excluded due to cognitive limitations. Three classes were assigned to the intervention group (IG, *n* = 61) and two classes to the control group (CG, *n* = 33). Further details are presented in Figure 1.

### 2.2 Instrumentation

#### 2.2.1 Motor Performance Tests

Maximal isometric strength, focusing on lower limbs, was assessed using the isometric mid‐thigh pull [25]. Explosive strength and jumping ability were measured using countermovement jumps [26]. Both tests were performed using a Leonardo Mechanograph force measurement platform (GRFP LT, 500 Hz, Novotec Medical, Pforzheim, Germany). Each child completed two attempts, with the better result used for analysis. Grip strength of the dominant hand was assessed using an automated handgrip dynamometer (Leonardo Mechanograph GF, 500 Hz; Novotec Medical, Pforzheim, Germany) while standing with the arm fully extended, as an indicator of overall strength [27]. All strength tests are valid and reliable in children (ICC = 0.86–0.93) [26, 28].

Speed and endurance were evaluated using a 20‐m sprint and a 20‐m shuttle run test, respectively. Sprint times were recorded with electronic time gates (WITTY, Microgate Srl, Bolzano, Italy, *r* = 0.90) [29]. The shuttle run followed a standardized protocol (start speed: 8 km/h) with progressive increases until two consecutive failures (*r* = 0.89) [5, 30].

Dynamic balance was evaluated by walking backwards on beams with three widths (6, 4.5, and 3 cm), with three attempts per beam and a maximum score of 72 points (ICC = 0.8) [31].

Coordination and explosive strength were assessed using the side hop test, which involves jumping sideways over a beam for 15 s, with the total number of correct jumps recorded from two trials (ICC = 0.95) [31].

Object control was assessed using an eye–hand coordination test involving repeated ball throws and catches over 30 s. The total successful catches from two trials served as the score (ICC = 0.87) [32].

#### 2.2.2 Attention and Health

Attention was assessed using a Flanker task, which primarily measures inhibitory control. The task was conducted in the classroom using E‐Prime 3.0 (PST, Pittsburgh, USA). Children received standardized instructions and identified the direction of a central target fish flanked by fish facing the same (congruent) or opposite (incongruent) direction [33]. Visual stimuli were presented for 300 ms with a 600–900 ms inter‐trial interval, and responses were allowed within 900 ms. After an eight‐trial practice block, children completed two test blocks of 36 trials. Reaction time, response accuracy, reaction time interference, and reaction time variability were recorded. The Flanker task is a valid and reliable measure of inhibitory control [34].

Physical health measures included height, body mass, body composition, and blood pressure. Body composition was assessed via bioelectrical impedance analysis (InBody270), showing high agreement with dual‐energy x‐ray absorptiometry (*r* ≥ 0.97) [35]. Blood pressure was measured using Omron M7 Intelli IT (Omron Healthcare, Kyoto, Japan) according to the European Society of Cardiology guidelines, with averaged values from repeated measurements used for analysis [36].

### 2.3 Procedures

Our study was a parallel, pragmatic, controlled trial involving 5 second‐grade classes from three socioeconomically diverse school centers in Basel, Switzerland. Socioeconomic background (SES) was determined using indicators from the *Sportcheck Study* [37]. Two classes were from a low‐SES area, one from a middle‐SES area, and two from a high‐SES area. Within each center, classes were assigned to either the IG or CG, with one additional class allocated to the IG.

Pre‐ and post‐assessments were conducted over 2 days. Motor performance was assessed in the gymnasium following a standardized 5 min warm‐up, while attention and health assessments were conducted on a separate day. The Flanker task was administered in the classroom with all children simultaneously, while the other tests were performed in a separate room for privacy. Feasibility in the IG was evaluated using simple visual rating scales assessing enjoyment and willingness to continue training.

Testing was performed by experienced staff. Children were familiar with most tests from previous Sportcheck assessments [5]. A single practice trial was provided for the mid‐thigh pull, grip strength, and countermovement jump (ICC = 0.91–0.93) [28]. No additional familiarization was performed due to demonstrated reliability and time constraints.

All children participated in three weekly 45‐min physical education lessons. The IG additionally completed 15 min of daily kettlebell‐based training (+75 min/week) over 7 weeks (October–December 2024). Training was conducted during regular class time and led by qualified instructors with a foundational qualification in children's sport and practical experience in youth physical activity. Training targeted the whole body using age‐appropriate strength and plyometric exercises organized by muscle groups across the week, with lower body training on Mondays and Thursdays, upper body on Tuesdays and Fridays, and core exercises on Wednesdays.

Only kettlebells (4–12 kg) and shock‐absorbing foam mats were used. Kettlebell selection was supervised by the instructors to ensure appropriate training loads. The mats served as jump targets, floor protection, and positioning aids to enhance safety. Children were instructed to position their feet next to the mat so that they remained outside the target zone if a kettlebell was released. All exercises were performed under continuous supervision to ensure safe and correct execution. Traditional, technically complex kettlebell movements were excluded, and training focused on simple exercises such as goblet squats, bent‐over rows, farmer carry, or high pulls. Strength exercises were performed for 1–3 sets of 10–20 repetitions, while plyometric exercises were completed for 20–30 s across 2–4 rounds.

Each session followed a consistent structure consisting of a 5 min cardiorespiratory warm‐up game, 5 min of strength and/or plyometric exercises, and a 5 min strength‐integrated game. Exercises were varied daily but deliberately kept low in complexity to ensure safe execution, emphasize fundamental movement patterns, and support the development of foundational strength and motor skills in children with no prior resistance training experience.

### 2.4 Data Analysis

Statistical analyses were conducted using R (Version 4.5.0, R Core Team 2025). Descriptive statistics (mean ± standard deviation) were calculated for all variables. To assess the effect of the intervention, a linear mixed model accounting for repeated measures was applied, with a particular focus on the time × group interaction. The model was adjusted for age and sex, and random intercepts were included. Participants were nested within classes, which were nested within schools, to account for the hierarchical structure of the data. Children with missing data at one of the two measurement time points were excluded from descriptive analyses. For inferential analyses, the mixed model approach is robust to missing data under the assumption of missing at random (MAR). By including random effects for participants, the model accounts for individual variability and provides reliable estimates despite incomplete observations.

This school‐based field study employed a pragmatic feasibility approach, in which recruitment focused on schools and classes willing to participate. Once a class consented, all children within that class were invited to participate, and only those providing informed consent were definitively included, allowing for a cluster‐based inclusion strategy. Sample size was therefore determined by class participation and individual consent rather than a priori power calculations, reflecting real‐world feasibility constraints in implementing school‐based interventions.

Interaction effects with confidence intervals (CIs) were reported for the raw data, Cohen's *d*, and percentage differences. Statistical significance was set at *p* < 0.05. Cohen's *d* was interpreted as small (*d =* 0.20–0.49), moderate (*d =* 0.50–0.79), and large (*d* > 0.80) [38]. It is important to note that for outcomes where a reduction indicates improvement, such as sprint time and reaction times in the Flanker task, the direction of change was inverted (i.e., multiplied by −1) during analysis. This adjustment was made to ensure that all positive effects consistently reflected performance improvements across outcomes.

## 3 Results

A total of 94 children were assigned to either the IG or CG. After the seven‐week intervention, 76 children (81.7% from baseline) participated in the post‐test. Dropouts resulted from injuries unrelated to the study (2 children), missing pre‐ or post‐test data (8 children), or insufficient training attendance (6 children). No adverse events related to the kettlebell training were reported. A maximum of 35 sessions could be conducted per class, but in two classes, scheduling conflicts reduced this to 33 sessions. Children were included in the analysis if they attended ≥ 80% of sessions. Mean attendance among the remaining participants was 91% (SD 5.5%). Baseline characteristics did not differ between groups and are presented in Table 1. Attrition analysis (not shown) revealed no meaningful differences between completers and dropouts regarding age, sex, or baseline motor performance. Of the 78 remaining participants, 47 (60.5%) were in the IG and 31 (39.5%) in the CG, yielding an IG:CG ratio of approximately 3:2. The training adherence in the IG group was 91% ± 5.5%.

**TABLE 1:** Baseline characteristics of participants.

	IG (*n* = 46)		CG (*n* = 31)	
Variable	Mean	SD	Mean	SD
Gender (female/male) (*n*)	24/22	—	18/13	—
Age (y)	7.8	0.4	7.9	0.5
Height (cm)	127.3	6.5	130.5	7.1
Weight (kg)	26.39	5.15	29.42	6.70
BMI Percentil (%)	0.52	0.33	0.60	0.31
BF (%)	19.46	8.19	21.97	8.33
MM (kg)	10.36	1.67	11.31	2.00
Adherence (%)	91.00	5.46	—	—

*Note:* Mean and standard deviation (SD).

Table 2 presents descriptive statistics (mean ± SD) for all outcome measures, while Table 3 summarizes the mixed model analysis, including raw data, *p*‐values, Cohen's *d*, and percentage changes, all presented in favor of the IG. A statistically significant effect was found for the sprint test (*p* = 0.01, *d =* 0.46, 95% CI [0.20–0.73]; Δ = +3.6%, 95% CI [1.5–5.6]). Non‐significant but small effect sizes with positive percentage changes were found for mid‐thigh pull (*p* = 0.136, *d =* 0.28 [−0.08 to 0.64]; Δ = +5.5% [−1.7 to 12.6]), eye–hand coordination (*p* = 0.25, *d =* 0.15 [−0.11 to 0.42], Δ = +8.0% [−5.7 to 21.8]), and the side hop (*p* = 0.48, *d =* 0.15 [−0.26 to 0.56], Δ = +4.4% [−7.6 to 16.4]). Given the wide CIs and non‐significant *p*‐values, these findings should be interpreted cautiously and cannot be considered indicative of a definitive intervention effect. Other motor performance outcomes showed negligible effects.

**TABLE 2:** Raw data from pre‐ and post‐assessments for CG and IG with mean and SD.

Variable	Pre	SD	Post	SD	Pre	SD	Post	SD
MM (kg)	10.37	1.66	10.71	1.71	11.31	2.00	11.55	2.16
BF (%)	19.66	8.21	20.30	9.81	21.97	8.33	22.29	8.38
SBP (mmHg)	98.17	7.05	96.63	9.56	99.80	8.59	99.57	10.35
DBP (mmHg)	66.70	6.71	65.61	8.36	67.23	8.31	65.83	8.89
Balance	36.98	14.16	38.36	16.11	31.77	13.44	35.35	14.53
EHC (*n*)	27.57	14.79	32.13	14.75	27.65	13.76	29.52	12.74
SH (*n*)	46.98	14.28	65.11	11.64	45.00	11.40	61.17	13.54
Sprint (*s*)	4.95	0.40	4.92	0.46	4.81	0.31	4.95	0.34
SRT (score)	3.94	1.76	4.13	1.89	3.58	1.48	3.97	1.76
GS (kg)	12.15	2.04	12.41	2.27	12.76	2.63	12.83	2.99
CMJ (m)	0.22	0.04	0.22	0.04	0.23	0.04	0.24	0.03
CMJ (W/kg)	31.08	4.51	31.89	4.33	33.64	4.53	34.57	3.97
MTP (kg)	52.55	10.76	60.65	13.69	56.02	10.40	60.08	14.19
RT con (ms)	564.43	70.96	542.36	49.28	560.65	57.94	532.04	63.10
Acc con (%)	0.75	0.15	0.90	0.09	0.67	0.27	0.85	0.15
RT inc. (ms)	572.49	74.41	550.66	54.83	578.56	75.78	541.21	54.46
Acc inc. (%)	0.72	0.15	0.88	0.08	0.67	0.20	0.84	0.16
RT inter. (ms)	8.06	33.48	8.30	28.81	17.89	47.72	9.16	40.48
RTSD (ms)	128.80	31.66	114.12	21.06	134.89	40.77	114.88	28.69

Abbreviations: BF, body fat; CMJ, countermovement jump; con, congruent; DBP, diastolic blood pressure; EHC, eye–hand coordination; GS, grip strength; inc., incongruent; inter, interference; kg, kilogram; *n*, number of Reps; m, meter; MM, muscle mass; mmHg, millimeters of mercury; ms, milliseconds; MTP, mid‐thigh pull; RT, reaction time; RTSD, reaction time standard deviation; s, seconds; SBP, systolic blood pressure; SH, side hop test; SRT, shuttle run test.

**TABLE 3:** Mixed model analysis assessing the intervention effects as raw data, Cohen's d and improvement in %.

	Raw (95%–KI)				Cohen's *d*			%		
	Time*GRP	INT LL	INT UL	*p*	Time*GRP	INT LL	INT UL	Time*GRP	INT LL	INT UL
MM	−0.03	−0.18	0.13	0.72	−0.02	−0.10	0.07	−0.3	−1.7	1.2
BF	−0.20	−1.60	1.20	0.78	−0.02	−0.19	0.14	−1.0	−7.8	5.8
Balance	−1.22	−6.02	3.59	0.62	−0.09	−0.43	0.26	−3.6	−17.6	10.5
EHC	2.20	−1.55	5.96	0.25	0.15	−0.11	0.42	8.0	−5.7	21.8
SH	2.00	−3.49	7.49	0.48	0.15	−0.26	0.56	4.4	−7.6	16.4
Sprint	−0.18	−0.28	−0.08	**0.001**	0.46	0.73	0.20	3.6	5.6	1.5
SRT	−0.15	−0.59	0.28	0.49	−0.09	−0.34	0.16	−4.0	−15.3	7.3
GS	0.06	−0.50	0.61	0.84	0.02	−0.22	0.27	0.5	−4.0	4.9
CMJ (m)	−0.01	−0.03	0.00	0.13	−0.31	−0.69	0.08	−5.7	−12.9	1.5
CMJ (W/kg)	0.13	−1.39	1.64	0.84	0.03	−0.30	0.35	0.4	−4.3	5.1
MTP	2.95	−0.89	6.79	0.14	0.28	−0.08	0.64	5.5	−1.7	12.6
SBD	−0.92	−4.81	2.97	0.64	−0.12	−0.62	0.38	−0.9	−4.9	3.0
DBD	0.51	−3.33	4.35	0.80	0.07	−0.46	0.60	0.8	−5.0	6.5
RT inc.	15.95	−17.44	49.35	0.35	−0.22	0.24	−0.68	−2.8	3.0	−8.6
RT con.	8.81	−23.40	40.99	0.59	−0.47	−0.01	−0.14	−5.4	−0.1	−1.6
RT inter	8.99	−15.30	33.27	0.47	−0.18	−0.01	−0.22	−57.59	−3.35	−72.57
RTSD	5.47	−9.65	20.59	0.48	−0.16	0.28	−0.59	−4.1	7.3	−15.6

*Note:* Adjusted for baseline age and sex.

Abbreviations: BF, body fat; CMJ, countermovement jump for height in meters (m) and relative power (W/kg); con, congruent; EHC, eye–hand coordination; GS, grip strength; inc., incongruent; INT, interval; inter, interference; LL, lower limit; MM, muscle mass; MTP, mid‐thigh pull; *p* value, in bold indicate a statistical significance **(*p* < 0.05)**; RT, reaction time; RTSD, reaction time standard deviation; SH, side hop test; SRT, shuttle run test; Time*Group Interaction term of time and group examines whether the change from pre‐ to post intervention differs between the intervention and control group; UL, upper limit.

No relevant changes were observed in muscle mass, body fat, systolic or diastolic blood pressure, or Flanker task outcomes. Detailed results are provided in Table 3.

A supplementary cluster analysis using intraclass correlation coefficients (ICCs) revealed that the observed outcomes were predominantly attributable to individual‐level variability, whereas class‐ and school‐level clustering accounted for only a negligible proportion of the variance. Detailed ICC values are provided in Table S1.

**FIGURE 2:**
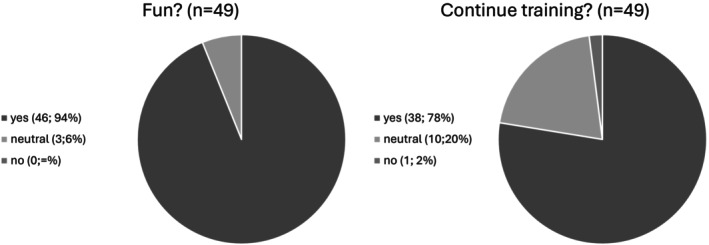
Survey of the IG on enjoyment and continuation of the training. *n* = number of children.

The survey results from the IG (Figure 2) showed that 46 of 49 children (94%, [87%–100%]) enjoyed the training, three (6%, [0%–13%]) were neutral, and none found it unenjoyable. Regarding training continuation, 38 children (78%, [65%–87%]) wanted to continue, 10 (20%, [11%–35%]) were indifferent, and one (2%, [0%–11%]) did not want to continue.

## 4 Discussion

The main objective of this study was to assess the feasibility and effectiveness of an innovative school‐based kettlebell program to improve motor performance, attention, and health in primary school children. Our seven‐week daily 15‐min classroom‐integrated intervention with second‐grade students was feasible and showed preliminary indications of effectiveness. The program was well‐tolerated across three schools from diverse rural socioeconomic areas. Children participated with high engagement and enjoyment, and teachers reported positive feedback. By integrating kettlebell training into regular classroom time without replacing physical education, we implemented a practical and time‐efficient approach to increasing daily physical activity that required only kettlebells and mats. Sessions were completed within approximately 20 min, minimizing classroom disruption, independent of weather, and did not require a change of clothing.

Feasibility was demonstrated by high acceptance among participants. 94% enjoyed the training, and 87% wished to continue. The intervention was easily implemented into daily routines, highlighting its potential for scalability in primary schools. Beyond feasibility, effectiveness was most evident in sprint performance, which improved significantly (*d =* 0.46, *p =* 0.01). Sprinting, a natural activity for children, is a reliable and valid measure of physical fitness in this population [29, 39], and the improvement aligns with previous research showing that strength training enhances sprint ability [40, 41]. To target explosive strength, plyometric elements were included, and although sprint‐specific training was limited by classroom space, sprint times improved, suggesting that daily short sessions can enhance lower body strength and speed [42, 43].

Small effect sizes were observed for the mid‐thigh pull (*d* = 0.28), side hop, and eye–hand coordination (each *d* = 0.15), but none of these results reached statistical significance and should be interpreted cautiously [44]. Mid‐thigh pull performance improved by 5.5%, consistent with its relation to sprinting and jumping, but variability in baseline performance and fixed bar height likely limited significance [28, 45, 46, 47, 48]. To our knowledge, this is the first school‐based intervention using the mid‐thigh pull to assess maximal isometric strength in children. This limits direct comparison with other studies, which typically use single‐leg hop outcomes [16, 24].

The side hop test reflects explosive strength and muscular endurance, but improvements were limited [16]. Although side hops were practiced regularly during the intervention, the observed effect size (*d* = 0.15) was smaller than expected. High coordinative demands and developmental constraints in younger children may explain this finding [49]. Nevertheless, the positive direction of change suggests that longer interventions may be required to elicit more pronounced effects.

There is no clear evidence linking strength training directly to eye–hand coordination. Object control skills, which underpin this test and are part of fundamental movement skills, are associated with higher levels of moderate‐to‐vigorous physical activity [50]. Our findings (*d* = 0.15) are consistent with this association. However, given the small, non‐significant effect size, no firm conclusions can be drawn.

Given the known links between mid‐thigh pull, sprinting, and jumping, the lack of improvement in countermovement jump performance was unexpected [46, 47]. Jumping involves coordination, technique, and stretch‐shortening cycle utilization, which can be challenging for children aged 6–9 [26, 45]. In comparison, the sprint test, as conducted in our study, closely resembles an activity that could naturally occur during children's recess on the playground. Given that motor coordination continues to develop with age, children aged 6–9 may be particularly challenged by complex coordination tasks, such as the countermovement jump [49]. This complexity may explain the inconsistent findings in the literature, with some studies showing improvements and others showing no improvements [42, 51]. Our training emphasized fast reactive jumps with short ground contact times rather than maximal height, which may have limited the transfer to countermovement jump performance. In untrained youth, specificity is key to measurable gains [52]. Additionally, children's neuromuscular characteristics, such as a lower rate of force development and limited type II fiber activation, reduce the explosive power, making short‐term improvements in countermovement jumps potentially less likely [53].

Similarly, no meaningful improvements were found in grip strength, which is commonly used as a proxy measure of overall strength [27]. This was somewhat unexpected, as training with kettlebells generally requires grip engagement. However, unlike conventional kettlebell programs that involve exercises such as swings, snatches, or Turkish get‐ups, our intervention focused on age‐appropriate movements [54]. As the children had no prior experience with strength training, the program focused on teaching basic movement patterns to avoid coordinative overload and ensure safe and age‐appropriate progression [49]. Kettlebells were used in various exercises as external loads, either carried as a bag or held in a front‐rack position, which minimized the effort for grip strength. However, grip strength involvement is limited in duration and intensity, and is likely insufficient to trigger measurable adaptations. This may explain the contrast with studies using more grip‐intensive protocols [55].

No meaningful improvements were observed in any of the remaining test outcomes. Possible explanations include an insufficiently specific training stimulus, as may have been the case for backward balancing and shuttle runs, which are primarily based on balance and endurance, respectively [56, 57]. Additionally, the use of a multicomponent approach rather than a targeted intervention may have limited effects on blood pressure [58]. Finally, considering the children's ongoing growth and development, substantial changes in body composition were not expected within such a short timeframe [18].

In addition to physical and health‐related outcomes, we assessed potential cognitive effects using the Flanker task, but no improvements were observed. Although regular physical activity can benefit executive functions [59, 60, 61], cognitive improvements are more likely in programs with sufficient physical and cognitive effort [62, 63]. It is possible that the kettlebell sessions provided insufficient cognitive stimulation, which could have contributed to the null findings. Cognitive engagement in training can be achieved through decision‐making, problem‐solving, accuracy, or memory tasks, which were not the focus of our intervention [64]. However, as training intensity was not monitored and the Flanker task was conducted under conditions limited by distractions and without a familiarization phase, these methodological limitations are a more likely explanation for the findings. Consequently, the validity of the null results is questionable, and they should not be overinterpreted.

### 4.1 Implications for School Health

Classroom‐integrated kettlebell training can be successfully implemented in primary schools when the following aspects are considered. Sessions should be brief (≈15 min) to fit within the school day and minimize disruption, yet frequent enough to foster habit formation and meaningful physiological adaptations [65]. Exercises need to be enjoyable to maintain engagement, but also safe and age‐appropriate, allowing for moderate‐to‐vigorous strength and conditioning work. Establishing simple routines for distributing, using, and storing equipment can enhance efficiency and adherence. Adhering to fundamental training principles ensures that the intervention provides a genuine training stimulus while remaining suitable for young children.

### 4.2 Limitations

A key limitation of this study was the lack of randomization. Five classes from three schools participated. In two schools, teachers chose whether their class was allocated to IG or CG, and the single class in the third school was automatically assigned to the IG. However, class‐ and school‐level clustering had negligible impact on the outcomes, with variability primarily at the individual level (see Table S1). The intervention was delivered by trained instructors, and all required equipment and resources were provided uniformly, minimizing potential variation due to teacher engagement or classroom resources. While SES distribution may still represent a potential source of bias, it was not assessed in this study and should be considered when interpreting the generalizability of the results. Another limitation is that physical activity outside of school (e.g., sports club participation) was not assessed, making it unclear whether the additional 15 min provided a substantial benefit or represented the children's main daily activity. Future studies should track overall activity with accelerometers. Training intensity also remains a significant limitation, as it was not directly monitored through heart rate. Consequently, the actual exertion level of the sessions is uncertain. Although sessions were designed to elicit moderate‐to‐vigorous activity, engagement likely varied, particularly with only one instructor supervising a single class. Training intensity is known to have a major influence on intervention effects, making accurate monitoring important. An alternative approach could have been to ask children to rate perceived exertion after each session using a Borg scale, but this would have substantially increased the time burden. In future studies, monitoring heart rate and/or collecting perceived exertion ratings would be valuable to determine the actual workload and better understand the effectiveness of the training. A further limitation concerns the Flanker task, which was conducted under conditions that were not fully controlled. All children completed the task simultaneously in their classroom (∼23 children with 2–4 teachers/study coordinators), which may have introduced distractions from neighboring classmates or personal items on their desks. Children who were still performing the task could also have been distracted by peers who had already finished. Additionally, no structured familiarization session was provided, which may have further impacted task performance [34]. Future studies should aim to minimize such distractions and include a familiarization phase to ensure a more reliable assessment.

Despite these limitations, strengths include the use of validated and reliable assessments, the high feasibility of the intervention, and its seamless integration into the classroom with minimal disruption. Requiring only kettlebells, the daily sessions provided both an effective training stimulus and a movement break. Importantly, the high frequency promoted routine and habit formation, key to sustainable health effects [65].

## 5 Conclusion

The classroom‐integrated kettlebell program was feasible and showed preliminary indications of effectiveness in second‐grade children. Daily 15‐min sessions led to measurable improvements in motor performance, particularly sprinting. The intervention demonstrates that brief, structured training can effectively enhance physical fitness in young children. Further long‐term studies are needed to assess sustained effects and inform educational and public health policies, supporting the integration of school‐based strength and fitness programs without compromising academic time.

## Funding

This work was supported by Starak.

## Ethics Statement

The pseudonymized data from this study cannot be made publicly available due to ethical and data protection requirements related to research involving children. The dataset includes sensitive physiological information that cannot be sufficiently de‐identified without risking participant confidentiality. The study was conducted in accordance with the Declaration of Helsinki and approved by the regional ethics committee (Ethics Committee Northwest and Central Switzerland, trial registration: NCT06910085), which did not permit unrestricted sharing of individual‐level data.

## Consent

Written informed consent was obtained from legal guardians allowing the use of anonymized data for scientific research under regulated conditions.

## Conflicts of Interest

The authors declare no conflicts of interest.

## Supporting information


**Table S1:** Intraclass correlation coefficients (ICCs) at the participant, class, and school levels.

## Data Availability

The data that support the findings of this study are available from the corresponding author upon reasonable request.
