# Supplementary material for: Classroom‐Integrated Kettlebell Training: Effects on Motor Performance, Attention, and Health in Primary School Children
Source: J Sch Health. 2026 Mar 19;96(4):e70136. doi: 10.1111/josh.70136 (PMC13001634; doi:10.1111/josh.70136)
Supplement: Supplementary file 1 — Table S1: Intraclass correlation coefficients (ICCs) at the participant, class, and school levels. [file JOSH-96-0-s001.docx]

Table 1: Intraclass correlation coefficients (ICCs) at the participant, class, and school levels.

|  | **ICC  (participant)** | **ICC  (class)** | **ICC  (school)** |
| --- | --- | --- | --- |
| **MM** | 0.97 | 0.00 | 0.01 |
| **BF** | 0.88 | 0.00 | 0.06 |
| **Balance** | 0.51 | 0.00 | 0.23 |
| **EHC** | 0.79 | 0.00 | 0.00 |
| **SH** | 0.52 | 0.00 | 0.04 |
| **Sprint** | 0.84 | 0.00 | 0.00 |
| **SRT** | 0.74 | 0.03 | 0.08 |
| **GS** | 0.86 | 0.00 | 0.00 |
| **CMJ [m]** | 0.61 | 0.00 | 0.00 |
| **CMJ [W/kg]** | 0.70 | 0.00 | 0.00 |
| **MTP** | 0.75 | 0.00 | 0.00 |
| **SBD** | 0.51 | 0.04 | 0.00 |
| **DBD** | 0.40 | 0.07 | 0.00 |
| **RT inc.** | 0.39 | 0.00 | 0.00 |
| **RT con.** | 0.30 | 0.00 | 0.01 |
| **RT inter.** | 0.00 | 0.00 | 0.00 |
| **RTSD** | 0.07 | 0.00 | 0.00 |

Values represent proportions of total variance derived from unconditional mixed-effects models including random intercepts for participant, class, and school. Class was used as a proxy for teacher effects. MM = muscle mass; BF = body fat; EHC = eye-hand coordination; SH = side hop test; SRT = shuttle run test; GS = grip strength; CMJ = countermovement jump for height in meters (m) and relative power (W/kg); MTP = mid-thigh pull; SDB = systolic blood pressure; DBD = diastolic blood pressure; RT = reaction time; con = congruent; inc = incongruent; inter = interference; RTSD = reaction time standard deviation.
